# Contemporary Pleural Drainage in Adult Practice: Image-Guided Techniques, Procedural Safety, Training Standards, and Malignant Pleural Effusion Management

**DOI:** 10.3390/jcm15145585

**Published:** 2026-07-16

**Authors:** Igor Barković, Stjepan Grgić, Tomislav Jakljević, Sandra Glavaš Kršul

**Affiliations:** 1 Clinical Hospital Center Rijeka, Krešimirova 42, 51000 Rijeka, Croatia; igor.barkovic@medri.uniri.hr (I.B.); tomislav.jakljevic@medri.uniri.hr (T.J.); 2 Faculty of Medicine, University of Rijeka, Braće Branchetta 20, 51000 Rijeka, Croatia; sgrgic@student.uniri.hr

**Keywords:** hemothorax, indwelling pleural catheter, lung ultrasound, pleural effusion, pleurodesis, pneumothorax, thoracic drainage, thoracoscopy

## Abstract

Pleural drainage is a fundamental procedure in the management of pneumothorax, pleural effusion, hemothorax, and complex pleural disease. Advances in imaging guidance, small-bore catheter technology, and structured training have improved the safety and effectiveness of pleural interventions. Despite its widespread use, pleural drainage continues to be associated with substantial complication rates, largely driven by operator inexperience, suboptimal insertion technique, and inadequate imaging support. Contemporary guidelines from the American Thoracic Society, European Respiratory Society, and British Thoracic Society emphasize evidence-based indications, routine ultrasound guidance, avoidance of trocar insertion, and the use of appropriately sized small-bore drains in most non-traumatic conditions. This review integrates current evidence and recommendations regarding indications, procedural techniques, imaging modalities, complication prevention, training standards, and malignant pleural effusion management into a practical framework for contemporary pleural drainage while highlighting areas of changing practice, persistent uncertainty, and key challenges in implementation.

## 1. Introduction

Pleural diseases represent a significant clinical burden, with an annual incidence exceeding 3000 cases per million population [1,2]. These conditions include spontaneous and traumatic pneumothorax, benign and malignant pleural effusions, pleural infection, parapneumonic effusions, and hemothorax, all of which may cause significant respiratory compromise if not promptly recognized and treated [3,4]. Chest tube thoracostomy remains a cornerstone intervention in pleural medicine and emergency practice. Although widely performed, complication rates of up to 20% have been reported, particularly when the procedure is undertaken without adequate training or imaging support [4,5,6,7].

Over the past decade, pleural practice has evolved considerably, with major advances in procedural safety and technique. These include the widespread adoption of point-of-care thoracic ultrasound (POCUS) to guide pleural interventions [4,5,8,9]; advances in the management of pleural infection, including risk-stratified drainage algorithms and intrapleural fibrinolytic therapy [10]; increased use of small-bore catheters and wire-guided (Seldinger) techniques, which reduce procedural pain and lower the risk of organ injury [3,11]; and clearer frameworks for the management of malignant pleural effusion, including patient-centered approaches, early use of indwelling pleural catheters (IPCs) and pleurodesis methods [3,12,13,14,15].

This review aims to integrate these developments into a single procedural and clinically oriented framework. This article examines pleural drainage as a shared intervention across respiratory medicine, thoracic surgery, emergency medicine, oncology, and intensive care. It synthesizes current evidence on patient selection, imaging-guided access, drain-size choice, insertion technique, digital drainage systems, complication prevention, training requirements, and malignant pleural effusion pathways. Particular emphasis is placed on areas where recommendations from different guidelines intersect, where clinical practice is changing, and where evidence remains limited or dependent on expert consensus. In this way, the review aims to support practical decision making and safer implementation of modern pleural drainage in everyday clinical practice.

## 2. Methods

This article is a narrative review of contemporary pleural drainage practice. A structured literature search was performed using PubMed/MEDLINE, Scopus, Web of Science, and the Cochrane Library. Additional relevant documents were identified through guideline repositories and the official websites of major professional societies, including the British Thoracic Society (BTS), European Respiratory Society (ERS), European Society of Thoracic Surgeons (ESTS), American Thoracic Society (ATS), Society of Thoracic Surgeons (STS), Society of Thoracic Radiology (STR), American College of Chest Physicians (ACCP), Eastern Association for the Surgery of Trauma (EAST), and other relevant surgical or emergency-medicine societies.

The literature search covered publications from January 1990 to December 2025. Because this was a narrative review, no strict date exclusion was applied to older landmark publications when they remained relevant to procedural safety, historical practice, or foundational clinical evidence. The final reference list included publications from 1993 to 2025, with priority given to contemporary guidelines, consensus statements, randomized trials, systematic reviews, meta-analyses, and clinically relevant observational studies published from 2010 onward, reflecting the major changes in image-guided pleural intervention, small-bore catheter use, intrapleural therapy, indwelling pleural catheters, digital drainage systems, and competency-based procedural training. The following search terms were used alone and in combination: “pleural drainage”, “chest tube”, “intercostal drain”, “tube thoracostomy”, “small-bore chest tube”, “large-bore chest tube”, “Seldinger technique”, “thoracic ultrasound”, “image-guided pleural procedure”, “pleural effusion”, “pleural infection”, “empyema”, “parapneumonic effusion”, “pneumothorax”, “hemothorax”, “malignant pleural effusion”, “indwelling pleural catheter”, “talc pleurodesis”, “digital chest drainage”, “persistent air leak”, “complications”, “training”, and “competence”.

Eligible publications included international guidelines, consensus statements, randomized controlled trials, systematic reviews, meta-analyses, prospective and retrospective cohort studies, clinically relevant observational studies, and major narrative reviews. Evidence was prioritized according to clinical relevance and methodological strength. Contemporary guideline documents and consensus statements were used as the primary framework where available. Randomized trials, systematic reviews, and meta-analyses were prioritized for specific clinical questions, while observational studies and expert reviews were included where randomized evidence was limited.

## 3. Indications for Pleural Drainage

Pleural drainage is a fundamental intervention used to evacuate air, fluid, or blood from the pleural cavity and restore physiological negative intrathoracic pressure [1,3]. Its indications include pneumothorax, pleural effusion, empyema, malignant pleural effusion, hemothorax, and postoperative thoracic management. Modern guidelines emphasize appropriate patient selection, imaging guidance, and the use of safer, minimally invasive techniques [3,4,5,10,14,15,16,17]. The indications for pleural drainage and the recommended drain size are summarized in Table 1.

### 3.1. Pneumothorax

Chest drain insertion is indicated in secondary spontaneous pneumothorax (SSP) with respiratory compromise, in large or symptomatic primary spontaneous pneumothorax (PSP), in traumatic pneumothorax, and in tension pneumothorax after initial needle decompression [3,4]. Small-bore catheters (≤14 Fr) are recommended as first-line treatment for most non-traumatic pneumothoraces according to the relevant international guidelines, including BTS, ERS/EACTS/ESTS, and ACCP statements [3,4,11,17,18]. Exceptions include patients receiving positive-pressure ventilation, those with large air leaks, and those with unstable physiology, in whom larger tubes may be preferable [16,19,20,21]. Ultrasound improves diagnostic speed, and its sensitivity for pneumothorax detection exceeds that of supine chest radiography, supporting its use in acute settings [5,9,22].

### 3.2. Pleural Effusion

Initial approach to pleural effusion consists of diagnostic (50 mL) and sometimes therapeutic pleural aspiration (maximum 1500 mL in one attempt), while chest tube drainage is indicated in large symptomatic effusions, complicated parapneumonic effusions, empyema and recurrent malignant effusions [3,4,10,11,14,15].

Chest tube insertion is indicated in pleural infection in the case of frankly purulent effusion (empyema) and in complicated parapneumonic effusions with pH ≤ 7.2, or a glucose concentration < 2.2 mmol/L and an elevated LDH > 1000 IU/L according to ERS/ESTS guidelines [10], or an LDH > 900 IU and glucose ≤ 4.0 mmol/L as recommended by BTS guidelines [3], and/or microorganisms detected on Gram stain or culture. If pH is >7.2 and <7.4, drainage should be considered when LDH is high, especially with ongoing fever, large fluid volume, low glucose, pleural contrast enhancement on CT, or septations and enclosed loculations on ultrasound [3,10]. If pH is ≥7.4, immediate drainage is generally not indicated. In some cases, patients with smaller parapneumonic collections, less than 5 cm on an erect lateral chest radiograph [23] or less than 2.5 cm on CT scan [24], can be successfully treated with antibiotic therapy alone, although with a mildly increased but statistically insignificant infection-related mortality [25]. The general practice of using large-bore chest drains (≥20 F) in the treatment of purulent and more viscous pleural effusions has significantly changed in recent years, as small-bore drains (≤14 F) are now endorsed as the first choice in most cases of pleural infections [3,10]. A retrospective analysis of the MIST-1 randomized controlled trial demonstrated no significant difference in primary (the combined frequency of death and surgery) and secondary outcomes (hospital stay, change in chest radiograph, and lung function at 3 months) in patients with pleural infection, related to the size of the chest drain used for their treatment, while pain scores were substantially higher in patients receiving large-bore drains, inserted by blunt dissection technique [26]. It is recommended to maintain the patency of a small-bore chest drain by regular flushing with 20–30 mL sterile saline every 6–8 h, as this has been shown to significantly reduce drain blockage by fibrin or pus [3,10,27]. Large-bore drainage may still be considered a pragmatic first option in selected situations, including postoperative pleural infection, empyema associated with a major air leak or bronchopleural fistula, recurrent obstruction of smaller catheters, and rare cases of esophageal-rupture-associated empyema, such as Boerhaave syndrome or iatrogenic perforation [28,29,30]. In the latter setting, a large-bore drain may be preferred to evacuate gastric contents together with pus while the patient is being prepared for urgent surgical management. However, these indications are supported largely by expert opinion and pathophysiological rationale rather than high-quality comparative evidence [28,29,30]. In the case of clinically significant pleural collection persisting after the initial chest drainage has ceased, the administration of intrapleural enzyme therapy (IET) containing intrapleural tPA and DNase is recommended [3,10], as combination IET has been shown to significantly improve the drainage of the infected collection, reducing the need for surgical referral and the length of hospital stay [31].

The approach to benign, non-infectious pleural effusions is primarily focused on the treatment of the underlying systemic disorder (e.g., heart failure, liver cirrhosis, renal failure, autoimmune diseases, and hypoalbuminemia). However, in the case of large symptomatic effusions when repeat therapeutic thoracentesis is required despite optimal medical therapy, chest drain insertion should be considered, while in selected cases talc pleurodesis, as well as IPC insertion may be performed [32,33].

### 3.3. Hemothorax

The aim of pleural drainage in hemothorax is to evacuate blood, monitor for ongoing bleeding, and to prevent blood clot formation, in order to achieve adequate lung re-expansion and prevent the development of fibrothorax in the long-term [11]. Traditionally, large-bore drains (≥28 Fr) have been used in the management of traumatic hemothorax due to high blood viscosity and tendency for clot formation [11]. However, more recent studies have shown that there is no significant difference in failure or complication rate between small- and large-bore tubes, with improved patient experience when using small-bore drains (≤14 Fr), including percutaneous pigtail catheters and smaller straight thoracostomy tubes [19,20,34,35,36]. The EAST guideline conditionally recommends a 14 Fr pigtail catheter, with ultrasound guidance for hemodynamically stable patients with traumatic hemothorax [37], while in hemodynamically unstable patients, emergent large-bore tube thoracostomy is the first choice [21,37]. Retained hemothorax increases the risk of empyema and fibrothorax, requiring early recognition and adequate evacuation by early video-assisted thoracoscopic surgery (VATS), ideally within 72 h when feasible, and generally within 4 days of diagnosis or initial drainage [21,37]. In the case of an elderly, fragile or critically ill patient unfit for surgical intervention, thrombolytics may be an effective alternative for retained hemothorax [37,38].

### 3.4. Postoperative and Iatrogenic Indications

Chest drains are routinely used after thoracotomy to prevent accumulation of air, blood and other fluids, as well as to promote lung re-expansion [39]. Digital drainage systems provide real-time monitoring of air-leak intensity and standardize criteria for drain removal, thereby improving postoperative recovery [40,41,42,43].

Iatrogenic pneumothorax is a relatively common complication of various diagnostic and therapeutic procedures, such as transthoracic needle lung biopsy, thoracentesis, pleural biopsy, bronchoscopy with transbronchial biopsy, pacemaker insertion, central venous catheterization, and positive pressure ventilation [44]. A small, asymptomatic iatrogenic pneumothorax in a stable, non-ventilated patient can often be observed, while immediate chest-tube insertion is indicated in large, symptomatic and tension pneumothoraces, as well as in mechanically ventilated patients due to the increased risk for tension pneumothorax and persistent air leak [45,46]. Iatrogenic hemothorax can also result from central venous catheterization, pleural intervention or transthoracic lung biopsy, where significant or symptomatic blood in the pleural space should be drained and output monitored [37]. Iatrogenic chylothorax can occur after cardiothoracic or esophageal surgery and after central venous access or pacemaker-related lymphatic/venous injury [47]. Pleural drainage may be required for symptomatic relief and lung re-expansion, although surgical intervention may be needed for persistent cases [48].

## 4. Imaging Guidance and Technological Advances

Imaging has become central to the safe and effective practice of pleural drainage, with strong evidence demonstrating that imaging-guided procedures significantly reduce complications, malposition rates, and procedural failure [3,5,9,22,49]. Modern pleural guidelines identify ultrasound as mandatory for nearly all non-emergency pleural interventions, reflecting a broader shift toward image-guided pleural practice [3,5]. The integration of digital drainage systems and CT-guided approaches has further advanced precision and safety in pleural medicine [40,41,42,43,49].

### 4.1. Role of Point-of-Care Ultrasound (POCUS)

Point-of-care ultrasound has become the preferred imaging modality for pleural interventions. It offers superior accuracy in identifying pleural fluid, distinguishing it from consolidated lung, detecting septations, and enabling real-time needle guidance. POCUS reduces the risk of organ injury, dry taps, and malposition and is associated with lower complication rates across multiple observational and randomized studies [5,9,22]. The ERS Statement on thoracic ultrasound Statement (2021) and BTS Clinical Statement on pleural procedures (2023) strongly recommend ultrasound use before pleural fluid procedures whenever feasible, particularly in elective and semi-elective settings, to identify the safest access site and reduce the risk of failed aspiration, organ injury, and drain malposition [4,5]. In emergency situations, such as tension pneumothorax, massive hemothorax, or severe physiological instability, immediate decompression or drainage should not be delayed in order to obtain imaging. Ultrasound demonstrates greater sensitivity than supine radiography for detecting pneumothorax and is recommended as the first-line modality in unstable patients [5,9,22]. Key sonographic signs include absence of lung sliding, the lung point, and the barcode sign on M-mode imaging [9,22]. These findings are particularly valuable in emergency, critical care, and post-procedural monitoring settings [9,19,22].

### 4.2. Role of Computed Tomography (CT) and Fluoroscopy

CT is more accurate than radiography for identifying tube malposition, kinking, intrafissural placement, and extrathoracic insertion [50]. It is especially useful when anatomy is distorted by prior surgery or malignancy, when complex or multiloculated effusions are present, or when complications such as empyema or bronchopleural fistula are suspected [51]. CT or fluoroscopy guidance for chest drain insertion may be required in cases of loculated pneumothorax with tethered lung, in the presence of bullae or posteriorly located pleural fluid collections, and when the sonographic view is limited [4,49].

### 4.3. Digital Drainage Systems

Digital chest drainage systems represent a major technological advance in pleural management, as they have several advantages over traditional water-seal systems. They provide real-time quantitative measurement of air leaks and display trends in air leaks over time, thus enabling the use of objective criteria for chest tube removal, as well as earlier identification of persistent air leaks or tube malfunction [39,52]. Modern digital drainage systems also provide standardized suction control to maintain the pre-set intrapleural pressure. Furthermore, they are compact and light and do not require attachment to the wall suction due to a built-in suction pump, resulting in earlier patient mobilization [39,52]. Several studies have demonstrated improved postoperative recovery and patient satisfaction, reduced drainage duration and length of hospital stay, reduced unnecessary delays in tube removal, and greater physician confidence in decision making when using digital drainage systems, particularly after lung resection [40,41,42,43].

### 4.4. Integration of Imaging and Technology

The combined use of POCUS, CT-imaging, and digital drainage systems is associated with fewer complications, faster clinical decision making, more accurate assessment of lung expansion, reduced rates of tube malposition and reintervention, and shorter hospital stays [3,5,9,22,39,40,41,42,43]. These advances form the core of modern pleural practice and are emphasized across all major contemporary guidelines [3,5,10,11,14,52].

## 5. Techniques of Pleural Drainage

### 5.1. Site of Insertion

The traditional site of intercostal drain insertion known as “the triangle of safety”, as described in the BTS 2003 guidelines [53], is defined by anatomical landmarks, as follows: the lateral border of pectoralis major anteriorly, anterior border of latissimus dorsi posteriorly, base of the axilla superiorly, and fifth intercostal space or horizontal line at the nipple-level inferiorly. The landmark technique is associated with higher rates of complications and malposition than imaging-guided methods [4,6,7,53]. Therefore, contemporary guidelines on the pleural procedures mandate the use of POCUS for all elective pleural fluid procedures to identify the safest site for drain insertion, as alternative sites may be more appropriate in the case of loculated collections or anatomic variations, when the safest access point lies outside the conventional triangle; the use of ultrasound significantly reduces the risk of organ injury, dry taps, and malposition [3,5,9,22].

### 5.2. Wire-Guided (Seldinger) Technique

The Seldinger technique is a percutaneous, wire-guided technique used for insertion of small-bore catheters (≤14 F), which reduces pain and minimizes soft-tissue trauma [3,4,35,52]. This method is preferred for benign and malignant pleural effusions, pneumothorax, and early-stage pleural infection [3,10]. Beyond conventional small-bore Seldinger placement, targeted wire-guided approaches may improve control over the final intrapleural position of the chest tube, regardless of its size. In a cadaver study by Protic et al. [54], targeted wire-guided large-bore chest tube placement with the use of their patent, a curved dilator, achieved a markedly higher success rate for correct tip positioning than the classical surgical technique (79% vs. 30%). A subsequent clinical study in patients with pneumothorax or pleural effusion likewise showed more accurate large-bore tube placement with the targeted wire-guided technique than with classical surgical insertion (78.4% vs. 36.6%), together with fewer overall post-procedural complications and less leakage of fluid along the drain [55]. This new method enabled more precise chest tube placement within the pleural cavity, improving the efficiency of thoracic drainage in terms of residual volume of air/fluid, malfunction of the chest tube and prolonged hospitalization [55]. Although these results are promising, the available evidence remains limited and reflects single-center experience rather than routine guideline-endorsed practice [54,55].

### 5.3. Blunt Dissection Technique

Large-bore tubes (≥20 F) are usually inserted by the blunt dissection technique [4,52]. The use of trocars for chest tube insertion is strongly discouraged due to the high rates of lung, cardiovascular, and intraabdominal injuries associated with this technique [4,6,52,56,57]. This approach is consistent with BTS and ATLS recommendations [3,4,56,57].

### 5.4. Choice of Chest Tube Size

The choice of chest tube size depends on the indication, as well as operator training and experience. Small-bore catheters are efficient in most cases of pneumothorax, malignant pleural effusion, other uncomplicated pleural effusions, and early empyema [3,10,11,12] and are associated with reduced pain and improved patient satisfaction [19,20,26]. The advantage of large-bore drains, however, is that they are less prone to kinking or blocking [4,52]. Large-bore drains are indicated in hemothorax in a hemodynamically unstable patient [3,19,20,34,35,36], in pneumothorax with large air leak [3,16,19,20,21] (secondary spontaneous pneumothorax, traumatic pneumothorax or patients on mechanical ventilation), in the treatment of empyema after failure of small-bore drains [3,10], and routinely after thoracic surgery [39]. Small-bore chest tubes can be used for ambulatory pleurodesis [58] and have shown to be effective although slightly inferior to large-bore drains in terms of pleurodesis achievement [15,59,60].

## 6. Complications and Risk Reduction

Despite being a routine intervention in respiratory, emergency, and surgical practice, pleural drainage carries a substantial risk of complications, with reported rates ranging from 8% to 20% in multicenter audits and observational cohorts [4,6,7]. The likelihood of complications is influenced by operator training, tube size selection and technique of insertion, imaging guidance, and the underlying pleural pathology [3,5,10]. Complications are typically categorized as insertion-related injuries, malposition and displacement, tube malfunction, infection, and problems arising from inadequate drainage [4,6,7]. Major international guidelines emphasize the use of thoracic ultrasound for pleural fluid procedures, avoidance of trocar insertion, appropriate site selection, and structured competency training to reduce preventable complications [3,5,10,16,61,62,63,64]. In emergency or trauma settings, imaging should be used when it is immediately available and clinically appropriate, but it should not delay life-saving decompression or drainage. Main pleural drainage complications and the strategies for risk reduction are summarized in Table 2.

### 6.1. Insertion-Related Injuries

Insertion-related injuries include lung laceration, intercostal vessel injury, diaphragmatic penetration, liver or splenic injury, and, in rare cases, cardiac or mediastinal injury. The risk increases significantly when large-bore tubes are inserted using anatomical landmarks alone, when trocars are used, and when operator experience is limited [4,6,7]. Ultrasound guidance reduces failed procedures, pneumothorax, and inadvertent organ puncture [5,9,22]. Trocars have largely been abandoned in modern pleural medicine [3,4,56,57]. Finger-sweep confirmation during blunt dissection is recommended to prevent intraparenchymal or subdiaphragmatic insertion [52,53].

### 6.2. Tube Malposition and Displacement

Malposition remains common even in experienced hands and includes intrafissural placement, intraparenchymal insertion, subcutaneous misplacement, and abdominal insertion [6,7,50]. Anteroposterior chest radiography detects fewer than one-third of malpositions, whereas ultrasound and CT are significantly more accurate [9,22,50,51]. CT is regarded as the diagnostic gold standard when malposition is suspected [49,50,51]. Displacement may occur because of inadequate fixation, traction on the drainage tubing and patient movement [4,61].

### 6.3. Tube Malfunction

Tube malfunction is typically caused by kinking, obstruction by fibrin, pus, or clots, blocked fenestrations, or inappropriate tube size [3,4,11]. Small-bore chest tubes are highly effective for pleural effusions and pneumothorax, but they may be less efficient in highly viscous empyema, organizing hemothorax, and multiloculated malignant effusion [4,10,11,19,62]. Digital drainage systems may assist in the early detection of poor drainage flow or tube obstruction [40,41,42,43].

### 6.4. Infection and Empyema

Procedural infections are associated with breaches in sterile technique, prolonged catheter dwell time, and inadequate drainage or source control of infected pleural collections [4,52,63]. Contemporary ERS/ESTS and BTS guidance emphasizes prompt drainage of established pleural infection, the use of appropriately sized small-bore drains in most cases, regular saline flushing to maintain drain patency, and intrapleural tPA/DNase when initial drainage has ceased but a residual infected collection remains [3,10,31,62].

### 6.5. Persistent Air Leak and Retained Collections

Persistent air leak (PAL) often occurs in secondary spontaneous pneumothorax, particularly in patients with underlying emphysema, as well as in mechanically-ventilated and postoperative thoracic-surgical patients [3,4,17,64]. Digital drainage systems improve PAL assessment by quantifying air-leak flow and helping guide the timing of tube removal [39,40,41,42,43]. Retained hemothorax is associated with an increased risk of empyema and may progress to fibrothorax or trapped lung; when post-thoracostomy drainage is incomplete, repeat imaging, preferably CT, and early VATS rather than placement of a second chest tube are recommended in stable patients [37,65].

## 7. Training, Competence, and Multidisciplinary Practice

### 7.1. Importance of Operator Experience

Operator experience is one of the strongest predictors of safety and success in pleural drainage. Multiple studies have shown that inadequate training significantly increases the risk of insertion-related complications, malposition, failed drainage, and the need for reintervention [4,66,67,68]. Pleural procedures performed by clinicians with limited experience are associated with higher rates of intercostal vessel injury, lung laceration, subcutaneous tube placement, organ injury, tube malfunction, and premature removal [4,6,7,66,67,68,69,70]. Contemporary pleural practice, therefore, emphasizes structured competency development, use of thoracic ultrasound for pleural fluid procedures, development of local safety standards and service models in which complex procedures are performed or supervised by experienced pleural operators [3,66,67,68,69,71].

### 7.2. Structured Training Programs

High-quality pleural training programs include didactic teaching in thoracic anatomy and pleural disease, hands-on simulation using mannequins or gel-based ultrasound trainers, supervised clinical procedures performed with real-time POCUS guidance, and competency assessment before independent practice [3,5,9,66,67,69,71]. Current evidence and guidance support simulation as an important element of competency-based training, as simulation-based education in pleural procedures improves procedural knowledge, technical performance, skill retention, and trainee confidence [66,69,72,73]. Contemporary BTS training standards also recommend direct observation of procedural skills (DOPS), detailed recording of procedural experience and complications, and competency-based progression, rather than procedure numbers alone [66,68].

### 7.3. Ultrasound Competence

Ultrasound competence is essential to modern pleural practice [3,4,5]. Operators must be able to identify pleural fluid and quantify effusion size, distinguish pleural fluid from atelectasis or consolidation, identify septations and pleural thickening, confirm safe insertion windows and rib spaces, and perform real-time needle guidance for smaller pleural loculations and pleural biopsies [5,66,69,74].

### 7.4. Multidisciplinary Pleural Services

Specialist pleural services and multidisciplinary pleural teams integrate expertise from respiratory/pleural physicians, thoracic ultrasound operators, thoracic surgery, radiology, pathology, oncology, palliative care, and specialist pleural nursing [3,75]. Such services support timely diagnosis, access to thoracic ultrasound and image-guided pleural intervention, ambulatory management of malignant pleural effusion, appropriate selection between IPC, pleurodesis, thoracoscopy, image-guided biopsy and surgery, and structured follow-up after pleural procedures [3,12,15,76]. Studies suggest that dedicated pleural services and MPE clinics can reduce hospital admissions, shorten inpatient stay, improve effusion control, reduce unnecessary or repeated procedures, and improve continuity of care [76,77].

### 7.5. Safety Culture and Documentation

A strong safety culture in pleural drainage includes standardized pre-procedure and post-procedure safety checks, such as confirmation of patient identity, laterality, consent, recent imaging, bleeding risk, equipment availability, appropriate sterile setup, clear documentation of the procedure and drain-management plan, post-procedure physiological monitoring, and ongoing assessment of drain function and patient symptoms [4]. BTS 2023 recommends completion of a safety checklist before and after all pleural procedures, and BTS Local Safety Standards for Invasive Procedures guidance emphasizes governance, good record keeping, team training, audit, reporting of adverse events, and a no-blame culture [3,78]. After chest drain insertion, a follow-up chest radiograph should be obtained within a few hours to confirm appropriate drain position [4]. Pleural checklists can reduce procedure-related complications and improve adherence to safety processes [79,80].

## 8. Pleural Drainage in Malignant Pleural Effusion

Malignant pleural effusion (MPE) is a frequent complication of advanced thoracic and extrathoracic malignancy and is associated with dyspnea, impaired quality of life, and high healthcare burden [12,81]. Management is primarily palliative and should focus on relief of breathlessness, avoidance of repeated pleural procedures, minimization of hospitalization, and alignment of treatment with patient priorities [3,14,15]. Contemporary BTS, ATS/STS/STR, and ERS/EACTS guidance supports an individualized approach in which therapeutic choice is guided by symptom burden, lung expandability, expected prognosis, performance status, patient preference, and local expertise [3,4,14,15].

### 8.1. Indications for Drainage in Malignant Pleural Effusion

Pleural drainage in malignant pleural effusion (MPE) is indicated in symptomatic, large effusion, while therapeutic intervention is generally not recommended for asymptomatic MPE [14]. In patients with suspected symptomatic MPE, initial ultrasound-guided thoracentesis can serve both as a diagnostic and therapeutic procedure, as it enables pleural fluid cytology sampling when malignancy is not yet confirmed, assessment whether dyspnea improves after fluid removal, and helps determine whether the lung re-expands sufficiently to make pleurodesis feasible [3,14,15].

**Figure 1:**
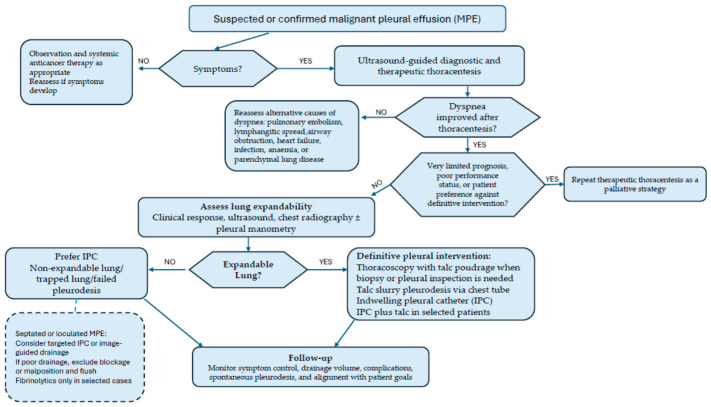
Algorithm for the management of malignant pleural effusion (MPE).

Recurrent symptomatic MPE should prompt consideration of a definitive pleural intervention rather than repeated thoracentesis alone, unless prognosis is very short, the patient is unfit for definitive treatment, or patient preference favors intermittent aspiration. The main definitive options are indwelling pleural catheter (IPC) and chemical pleurodesis, most commonly with talc [3,12,14,15,81]. Assessment of lung expandability is central before selecting pleurodesis. Failure of lung re-expansion after drainage suggests a non-expandable lung, in which pleurodesis is unlikely to succeed and IPC is generally preferred. Lung expansion can be assessed clinically by ultrasound, particularly M-mode assessment of the sinusoidal sign and atelectatic lung movement, and radiography after therapeutic drainage, whereas pleural manometry may provide additional information in selected cases [3,8,12,14,15,76]. We provide the algorithm for clinical decision making in the management of MPE in Figure 1.

### 8.2. Chemical Pleurodesis

Chemical pleurodesis is indicated in symptomatic MPE, when the lung is expandable after drainage, and the patient is suitable for a definitive pleural intervention [3,14,15]. The aim is to achieve adhesion between the visceral and parietal pleura by inducing inflammation and scarring, thereby preventing recurrent fluid accumulation. Adequate pleural apposition is crucial for pleurodesis success. Talc remains the best-established sclerosant for MPE and can be administered either as talc slurry through a chest tube or as talc poudrage during thoracoscopy [3,14,15,82]. Medical-grade talc should be sterile, asbestos-free and large-particle (graded), since systemic inflammation and acute respiratory distress syndrome were mainly linked to ungraded or small-particle talc preparations and/or high doses [15,83,84]. Other sclerosants, such as doxycycline, minocycline, bleomycin, silver nitrate, and iodopovidone, have been studied, but their evidence base, availability, toxicity, cost or tolerability are generally less favorable than talc [15]. A Cochrane analysis demonstrated that talc slurry resulted in fewer pleurodesis failures than bleomycin or doxycycline, while talc poudrage was likely to be similarly effective to talc slurry [82]. For this reason, modern international guidance and most contemporary trials focus primarily on talc-based pleurodesis, with alternative agents generally reserved for settings in which talc is unavailable or unsuitable.

#### 8.2.1. Talc Slurry

Talc slurry involves instillation of sterile talc suspended in saline through an intercostal chest tube after pleural drainage and confirmation of adequate lung re-expansion. This method is less invasive than thoracoscopy, can be performed at the bedside or in a procedural unit, and is applicable in patients who do not require thoracoscopic biopsy or who are unsuitable for thoracoscopy [3,12,14,15,81]. Small-bore drains are commonly used for talc slurry pleurodesis because they are less invasive and generally better tolerated than large-bore surgical drains [4]. BTS 2023 states that small-bore drains are associated with less pain during insertion and while in situ, but also notes that large-bore drains are less likely to kink or block [3,4]. Evidence on pleurodesis efficacy by drain size is mixed: the TIME1 randomized trial found that 12F tubes caused a statistically significant but clinically modest reduction in pain compared with 24F tubes, but failed to meet non-inferiority criteria for pleurodesis efficacy [60]. In contrast, a later meta-analysis of randomized trials concluded that small- and large-bore tubes had similar pleurodesis success and complication rates [59]. Therefore, small-bore drains remain a reasonable and widely used option when talc slurry pleurodesis is selected, however, BTS recommends chest tubes inserted with the plan of subsequent talc-slurry pleurodesis should be at least 12F in diameter, and possibly larger, to avoid blockage by talc particles [4].

#### 8.2.2. Talc Poudrage

Talc poudrage is performed during local anesthetic medical thoracoscopy or surgical thoracoscopy (VATS). It allows pleural fluid evacuation, direct visual inspection of the pleura, targeted pleural biopsies and insufflation of talc under during the same procedure [3,14,15]. It is particularly suitable when a histological diagnosis or additional tissue for molecular testing is needed and when local expertise in thoracoscopy is available. The relative efficacy of poudrage and slurry remains debated. A study by Dresler et al., which included 501 patients with MPE, found no overall difference, but in post hoc analysis, talc poudrage was superior to talc slurry in terms of pleurodesis success (82% vs. 67%) in the lung-cancer- and breast-cancer-patient subgroups [85]. However, the rate of adverse events, including respiratory complications, respiratory failure, and death, was also higher in the poudrage group [85]. In a more recent comparison, the TAPPS randomized clinical trial in 330 patients with MPE, thoracoscopic talc poudrage did not significantly reduce pleurodesis failure compared with talc slurry via chest tube at 90 days: failure occurred in 22% of the poudrage group versus 24% of the slurry group [86]. No statistically significant differences were found in prespecified secondary outcomes. The results of a 2024 meta-analysis of talc slurry vs. talc poudrage for pleurodesis found both methods to be similar in efficacy and safety [87]. The advantages of poudrage are diagnostic and procedural integration: it permits direct pleural inspection, biopsy, and pleurodesis in a single procedure. However, its availability is limited as it requires thoracoscopy facilities, trained operators, sedation or anesthetic support and a medically fit patient for the procedure.

### 8.3. Indwelling Pleural Catheters (IPCs)

Significant non-expandable lung reduces the likelihood of successful pleurodesis, and BTS guidelines suggest that IPC should be considered rather than talc pleurodesis when more than 25% non-expandable lung is present [3]. IPCs allow intermittent outpatient or home-based drainage, avoid the need for prolonged inpatient chest-tube drainage, and are particularly useful when recurrent fluid accumulation is expected. Contemporary guidelines support an individualized choice between IPC and chemical pleurodesis in patients with symptomatic MPE and expandable lung, based on expected prognosis, performance status, and patient preference [3,14,15]. IPCs are especially valuable in patients with non-expandable lung, failed pleurodesis or loculated MPE. Compared with inpatient talc pleurodesis, IPC treatment provides similar relief of breathlessness and similar quality-of-life improvement, while the main advantages of IPC are shorter initial hospital stay, fewer subsequent inpatient days, and fewer repeat invasive pleural procedures, although IPCs were associated with more cellulitis than chemical pleurodesis [88,89,90]. Spontaneous pleurodesis may occur during IPC treatment, allowing catheter removal in up to 45% of patients [91,92]. Daily drainage can increase spontaneous pleurodesis compared with less frequent or symptom-guided drainage [93].

#### Combination Therapy: IPC Plus Talc Instillation

Talc instillation through an indwelling pleural catheter can be performed in selected patients with symptomatic MPE who have an IPC in place, adequate lung expansion after drainage, and no substantial non-expandable lung [3,14,15,94]. The strongest evidence comes from the IPC-PLUS randomized placebo-controlled trial, in which patients underwent IPC insertion and initial outpatient drainage, followed by instillation of talc or placebo through the IPC [94]. At day 35, successful pleurodesis occurred in 43% of patients in the talc group compared with 23% in the placebo group; at day 70, successful pleurodesis occurred in 51% versus 27%, respectively [94]. The main advantage of IPC plus talc is that it may increase the chance of pleurodesis and earlier catheter removal while preserving outpatient management.

### 8.4. Septated and Loculated MPE

Septated or loculated MPE is challenging because percutaneous drainage may not evacuate all fluid, and incomplete lung expansion reduces the likelihood of successful pleurodesis [3,15]. IPC should be considered when symptomatic loculated MPE coexists with non-expandable lung or failed pleurodesis, provided the catheter can access the symptomatic fluid compartment [3,14]. If IPC drainage becomes impaired, catheter blockage or malposition should be excluded and saline or heparinized saline flushing attempted [3,95]. Intrapleural fibrinolytics may be considered in highly selected symptomatic patients, particularly when IPC-related loculation persists despite flushing, but evidence remains limited and routine use is not recommended [3,96,97]. Surgical intervention is reserved for a minority of patients with significant symptoms and favorable performance status and prognosis [3,14,15].

### 8.5. Complications of MPE Drainage

Catheter blockage or poor drainage may result from fibrin, kinking, malposition, tumor progression, septations, or loculations. Initial management includes clinical assessment, imaging when needed, and flushing with saline or heparinized saline. In selected symptomatic patients with IPC-related loculation, intrapleural fibrinolytics may be considered, but evidence is limited and routine use is not recommended [3,95,96,97]. In malignant pleural effusion, IPC-related infections are uncommon, occurring in approximately 5–6% of patients in large studies and meta-analyses, although reported series vary more widely [63,90]. Most IPC infections can be managed successfully with antibiotics, often without catheter removal, but close clinical monitoring is required [14,63,90]. Superficial cellulitis is usually managed with oral antibiotics, while deep pleural infection requires systemic antibiotics and continued drainage, with catheter removal reserved for persistent sepsis, failed treatment, or catheter malfunction [14,63,90,95]. Pain during drainage may occur, especially with rapid fluid evacuation, excessive negative pressure, or non-expandable lung. Management includes slowing or stopping drainage, reducing drainage volume, analgesia, and reassessing lung expandability if symptoms recur [95]. Catheter-tract metastasis is uncommon but recognized, particularly in mesothelioma or heavy pleural tumor burden. It presents as painful nodularity along the catheter tract and is usually managed palliatively with analgesia, radiotherapy, systemic treatment, or catheter removal when necessary [95,98].

## 9. Future Directions in Pleural Medicine

Current research in pleural medicine is moving toward more individualized, ambulatory, and competency-based care. A further area of development is the integration of prognostic models into pleural pathways. In pleural infection, validated risk-stratification tools such as the RAPID score, together with emerging biomarker, microbiological, and ultrasound-based predictors, may help identify patients at higher risk of poor outcome or need for treatment escalation, although prospective studies are still required before these models can reliably guide early surgery or intrapleural therapy [3,10,99,100]. In malignant pleural effusion, the LENT score was the first validated prognostic score for survival prediction, while the PROMISE score incorporated clinical variables and pleural fluid biomarkers to estimate 3-month mortality. More recent external validation data suggest that LENT and PROMISE retain prognostic value, although their routine uptake in clinical practice remains variable [99,100]. These models may help guide discussions regarding prognosis, treatment burden, ambulatory versus inpatient management, and appropriateness of definitive pleural interventions. However, prognostic scores should be used as adjuncts to clinical judgement and multidisciplinary decision making rather than as standalone determinants of care. In malignant pleural effusion, future priorities include optimizing patient selection between IPC, talc pleurodesis, thoracoscopy, and combined approaches; refining the timing and route of talc administration; and evaluating novel pleurodesis strategies [4,12,14,15,86]. Ambulatory pleural pathways, particularly IPC-based management and early referral from oncology to specialist pleural services, are increasingly important as they reduce hospital dependence, effusion-related admissions and repeat pleural procedures [12,76,77,94]. Further development of thoracic ultrasound certification, structured pleural-procedure training, DOPS-based assessment and simulation-supported education is likely to improve procedural safety and standardize competence across specialties [66,69,74]. Overall, these trends point toward pleural care that is increasingly patient-centered, minimally invasive, and outpatient-oriented.

## 10. Conclusions

Pleural drainage remains a fundamental intervention across respiratory medicine, thoracic surgery, emergency medicine, oncology, and intensive care. Safe and effective practice depends on appropriate patient selection, accurate imaging assessment, individualized drain-size selection, meticulous insertion technique, structured post-insertion management, and timely escalation when drainage fails.

Thoracic ultrasound should be used for pleural fluid procedures whenever feasible, particularly in elective and semi-elective settings, while emergency decompression or drainage should not be delayed in unstable patients. Small-bore image-guided drains are suitable for many stable non-traumatic pleural conditions, whereas large-bore drains retain an important role in selected traumatic, postoperative, unstable, high-output, major air-leak, or recurrent-obstruction scenarios.

Complication prevention requires avoidance of trocar insertion, careful site selection, secure drain fixation, regular assessment of drain function, and early recognition of malposition, blockage, persistent air leak, or retained collections. These principles should be supported by competency-based training, simulation, thoracic ultrasound education, procedural checklists, audit, and multidisciplinary pleural pathways.

In malignant pleural effusion and other complex pleural conditions, drainage should be integrated into patient-centered decision making based on symptoms, lung expandability, prognosis, performance status, patient preference, and local expertise. The goal of modern pleural drainage is, therefore, not simply evacuation of air or fluid, but safe, durable symptom control with the lowest possible burden of pain, complications, hospitalization, and repeat procedures.

## Figures and Tables

**Table 1:** Indications for Pleural Drainage and the Preferred Drain Size.

Clinical Indication	BTS Recommendations	ERS/ESTS/EACTS Recommendations	ATS/STS/STR Recommendations	ACCP Recommendations	Preferred Drain Size/Technique	Imaging Guidance	Special Considerations
Primary or secondary spontaneous pneumothorax, clinically stable	Supports less invasive management and small-bore drainage in appropriate non-traumatic pneumothorax; management depends on symptoms, size, and physiological compromise	ERS/EACTS/ESTS guidance supports individualized management and less invasive approaches in suitable stable patients	Not primarily addressed in ATS/STS/STR MPE guidance	ACCP guidance supports intervention in larger or symptomatic pneumothoraces and recognizes catheter drainage as an option in appropriate patients	Small-bore catheter, usually ≤14F, is appropriate for most stable non-traumatic pneumothoraces	Chest radiography is commonly used; ultrasound may support diagnosis in experienced hands; CT in selected complex cases	Larger drains may be considered in major air leak, unstable physiology, mechanical ventilation, or traumatic pneumothorax
Traumatic pneumothorax or pneumothorax with major air leak	BTS principles support individualized drain choice where large air leak or instability is present	ERS pneumothorax guidance is less directly applicable to traumatic pneumothorax; clinical stability remains central	Not applicable	ACCP spontaneous pneumothorax guidance is less directly applicable to traumatic pneumothorax	Drain size should be individualized; larger drains may be preferred with major air leak, mechanical ventilation, or physiological instability	Ultrasound is useful in acute trauma assessment when immediately available; CT when clinically appropriate and patient stability allows	Tension pneumothorax requires immediate decompression followed by definitive pleural drainage; imaging should not delay emergency treatment
Benign non-infectious pleural effusion requiring drainage	Supports ultrasound-guided pleural fluid procedures and small-bore drainage when chest tube drainage is required	ERS benign pleural effusion guidance emphasizes treatment of the underlying cause, with drainage reserved for selected symptomatic cases	Not applicable	Not a main focus of ACCP chest tube size recommendations	Small-bore image-guided catheter, usually ≤14F	Thoracic ultrasound should be used for pleural fluid procedures whenever feasible	Treat the underlying systemic disorder; repeated aspiration, pleurodesis, or IPC may be considered only in selected recurrent symptomatic cases
Pleural infection/complicated parapneumonic effusion/empyema	Recommends small-bore chest tube drainage as initial drainage for pleural infection; regular flushing to maintain patency	ERS/ESTS statement supports small-bore image-guided drainage as first-line in most adult pleural infections	Not applicable	ACCP classification helps identify parapneumonic effusions requiring drainage, but modern tube-size guidance is better addressed by BTS and ERS/ESTS	Small-bore drain, usually ≤14F, first-line in most cases	Ultrasound to identify septations and safe access site; CT in complex, loculated, postoperative, or non-resolving cases	Maintain patency with saline flushing; consider tPA/DNase when residual infected collection persists; early surgical input if drainage fails
Selected complex empyema scenarios	Supports escalation when medical drainage fails; small-bore first-line does not exclude selected large-bore use	ERS/ESTS emphasizes individualized escalation, intrapleural therapy, and surgery where appropriate	Not applicable	Less specific for modern tube-size selection in complex empyema	Large-bore drainage may be considered pragmatically in selected cases	Ultrasound or CT depending on anatomy and collection location	Examples include postoperative empyema, major air leak or bronchopleural fistula, repeated small-bore catheter obstruction, and oesophageal-rupture–associated empyema; evidence is mainly expert opinion
Stable traumatic hemothorax/hemopneumothorax	Not primarily addressed in BTS pleural disease guidance	Not primarily addressed in ERS pleural disease guidance	Not applicable	Not applicable	14F pigtail catheter may be considered in haemodynamically stable patients according to trauma literature and EAST guidance	Ultrasound may be used; CT is useful for retained hemothorax	Monitor output and clinical stability; retained hemothorax should prompt CT reassessment and early VATS rather than repeated blind tube placement
Unstable traumatic hemothorax or high-output hemothorax	Not primarily addressed in BTS pleural disease guidance	Not primarily addressed in ERS pleural disease guidance	Not applicable	Not applicable	Emergent large-bore tube thoracostomy is generally preferred	Imaging should not delay emergency treatment in unstable patients	Large-bore drainage allows rapid evacuation of blood and monitoring of ongoing bleeding; operative management may be required depending on instability and output
Postoperative thoracic drainage	BTS pleural procedures guidance emphasizes safe insertion technique, avoidance of trocar insertion, and appropriate post-procedure care	ERAS/ESTS guidance supports optimized postoperative drainage pathways and digital drainage systems	Not applicable	Not applicable	Drain size depends on procedure, expected air leak, bleeding risk, and surgeon preference; larger drains remain common after thoracic surgery	Postoperative radiography and/or digital drainage monitoring	Digital systems allow objective air-leak assessment and may support earlier drain removal; management should follow local thoracic surgical protocols
Malignant pleural effusion requiring talc slurry pleurodesis	Supports definitive intervention for symptomatic recurrent MPE; for talc slurry, BTS recommends a drain of at least 12F, and possibly larger, to reduce blockage risk	ERS/EACTS supports individualized choice between IPC, talc pleurodesis, and thoracoscopy according to lung expansion, prognosis, preference, and expertise	ATS/STS/STR supports individualized choice between IPC and pleurodesis in symptomatic recurrent MPE with expandable lung	Not applicable	Small-bore drains are commonly used; avoid very small catheters when talc slurry is planned	Ultrasound-guided insertion; radiography or ultrasound to assess lung expansion	Pleurodesis requires adequate lung expansion and pleural apposition; thoracoscopy with talc poudrage may be preferred when biopsy or direct pleural inspection is needed
Malignant pleural effusion with non-expandable lung, failed pleurodesis, or preference for ambulatory care	Supports IPC, especially in substantial non-expandable lung or failed pleurodesis	ERS/EACTS supports IPC in non-expandable lung and as part of individualized MPE care	ATS/STS/STR supports IPC for non-expandable lung, failed pleurodesis, or loculated effusion	Not applicable	Indwelling pleural catheter	Ultrasound-guided insertion	IPC is generally preferred for non-expandable lung, failed pleurodesis, or outpatient symptom control; septated/loculated MPE may require targeted drainage

**Table 2:** Major Complications of Pleural Drainage and Prevention Strategies.

Complication	Main Risk Factors	Prevention/Mitigation Strategy
Organ injury, including lung, diaphragm, liver, spleen, heart, or mediastinal injury	Landmark-based insertion, trocar use, distorted anatomy, low insertion site, operator inexperience	Use ultrasound guidance for pleural fluid procedures; avoid trocar insertion; select the safest site; use CT guidance in complex anatomy; ensure adequate supervision and training
Intercostal vessel injury/bleeding	Incorrect insertion plane, coagulopathy, anticoagulant or antiplatelet therapy, traumatic insertion	Assess bleeding risk; correct reversible coagulopathy when possible; insert immediately above the rib; avoid unnecessary force; use imaging guidance where appropriate
Tube malposition	Emergency insertion, lack of imaging guidance, adhesions, fissural placement, obesity, loculated collections	Use ultrasound or CT guidance when needed; confirm position after insertion; consider CT if drainage is poor or malposition is suspected
Tube displacement	Poor fixation, patient movement, traction on tubing, inadequate dressing	Secure the drain carefully; apply appropriate dressing; avoid traction; educate staff and patient; document drain position and external length
Tube blockage or kinking	Small lumen, viscous pus, fibrin, blood clot, talc particles, inadequate flushing	Choose appropriate drain size for the indication; avoid dependent kinking; flush small-bore drains regularly in pleural infection; consider larger drain or additional image-guided drainage if repeated obstruction occurs
Persistent infected collection	Loculated empyema, inadequate drain position, drain blockage, delayed drainage	Reassess with ultrasound or CT; ensure drain patency; use intrapleural tPA/DNase when indicated; involve thoracic surgery early if drainage fails
Retained hemothorax	Clotted blood, delayed drainage, insufficient evacuation	Monitor output and imaging; use CT when retained collection is suspected; consider early VATS, ideally within the early post-injury window when feasible
Persistent air leak	Secondary spontaneous pneumothorax, emphysema, mechanical ventilation, postoperative air leak, bronchopleural fistula	Use appropriate drain and suction strategy; consider digital drainage to quantify air leak; involve thoracic surgery or interventional pulmonology if persistent
Procedure-related infection	Breaks in sterile technique, prolonged drain duration, poor catheter care	Use full aseptic technique; secure and dress the drain properly; remove the drain as soon as clinically appropriate; treat established IPC or drain infection with antibiotics and continued drainage where possible
Re-expansion pulmonary oedema or drainage-related pain	Rapid evacuation of large effusion, excessive negative pressure, non-expandable lung	Avoid overly rapid large-volume drainage; stop or slow drainage if chest pain, cough, or instability occurs; reassess lung expandability
Subcutaneous emphysema	Poor drain position, side holes outside pleural cavity, persistent air leak, drain blockage	Confirm side-hole position; ensure tube patency; avoid kinking; manage persistent air leak appropriately
Catheter-tract metastasis after IPC or pleural intervention	Mesothelioma or heavy pleural tumour burden	Recognize painful tract nodularity; manage palliatively with analgesia, radiotherapy, systemic treatment, or catheter removal when necessary

## Data Availability

No new data were created or analyzed in this study. Data sharing is not applicable to this article.

## Abbreviations

The following abbreviations are used in this manuscript:

ACCP	American College of Chest Physicians
ATS	American Thoracic Society
ATLS	Advanced Trauma Life Support
CT	Computed tomography
DNase	Deoxyribonuclease
DOPS	Direct observation of procedural skills
EACTS	European Association for Cardio-Thoracic Surgery
EAST	Eastern Association for the Surgery of Trauma
ERS	European Respiratory Society
ESTS	European Society of Thoracic Surgeons
IET	Intrapleural enzyme therapy
IPC	Indwelling pleural catheter
LDH	Lactate dehydrogenase
LENT	pleural fluid LDH, ECOG performance status, blood Neutrophil-to-lymphocyte ratio, Tumor type
MIST1	The First Multicenter Intrapleural Sepsis Trial
MPE	Malignant pleural effusion
PAL	Persistent air leak
POCUS	Point-of-care ultrasound
PROMISE	PROgnostic score for Malignant pleural effuSion
PSP	Primary spontaneous pneumothorax
RAPID	Renal, Age, Purulence, Infection source, Dietary factors Score
SSP	Secondary spontaneous pneumothorax
STR	Society of Thoracic Radiology
STS	Society of Thoracic Surgeons
TAPPS	Evaluating the efficacy of Thoracoscopy And talc Poudrage versus Pleurodesis using talc Slurry trial
TIME1	First Therapeutic Intervention in Malignant Effusion Trial
tPA	Tissue plasminogen activator
VATS	Video-assisted thoracoscopic surgery
